# Acetylated Polysaccharides From *Pleurotus geesteranus* Alleviate Lung Injury Via Regulating NF-κB Signal Pathway

**DOI:** 10.3390/ijms21082810

**Published:** 2020-04-17

**Authors:** Xinling Song, Jianjun Zhang, Jian Li, Le Jia

**Affiliations:** 1 College of Food and Biological Engineering, Jimei University, Xiamen 361021, China; 2 College of Life Science, Shandong Agricultural University, Taian 271018, China

**Keywords:** Acetylated polysaccharides, Acute lung injury, Antioxidant, *Pleurotus geesteranus*

## Abstract

The present work investigated the anti-inflammatory, antioxidant, and lung protection effects of acetylated *Pleurotus geesteranus* polysaccharides (AcPPS) on acute lung injury (ALI) mice. The acetylation of AcPPS was successfully shown by the peaks of 1737 cm^−1^ and 1249 cm^−1^ by FTIR. The animal experiments demonstrated that lung damage can be induced by zymosan. However, the supplementation of AcPPS had potential effects on reducing lung index, remitting inflammatory symptoms (TNF-α, IL-1β, and IL-6), inhibiting NF-κB signal pathway based on up-regulating the level of IκBα and down-regulating p-IκBα level by Western blotting and immunofluorescence assay, preventing oxidative stress (ROS, SOD, GSH-Px, CAT, T-AOC, and MDA), reducing lipid accumulation (TC, TG, LDL-C, HDL-C, and VLDL-C), and alleviating lung functions by histopathologic observation. These results demonstrated that AcPPS might be suitable for natural food for prevention or remission in ALI.

## 1. Introduction

Systemic inflammatory response syndrome (SIRS) is caused by the body’s systemic uncontrolled inflammatory immune response due to a variety of severe stimulations or attacks including infection, hypoxia, trauma, necrosis, and reperfusion [1]. As a result of excessive inflammation, it is a serious disease with complex etiology and serious condition, resulting in acute organ injury, shock, and multiple organ dysfunction syndrome (MODS) [2], while acute lung injury (ALI) is the earliest, has the highest incidence, and is also one of the important causes of death [3]. On the account of the lung being the most vulnerable target organ when SIRS occurs, ALI is a severe inflammatory disease caused by uncontrolled oxidative stress and inflammatory process [4].

Goris [5] first described a model of zymosan-induced generalized inflammation (ZIGI) in mice which resembled human MODS. The ZIGI model is widely used to research the processes leading to organ injury. A large number of studies have confirmed that intraperitoneal injection of zymosan can induce lung injury, furthering to build ALI model [6]. Zymosan (ZY) is a substance extracted from the cell wall of *Saccharomyces cerevisiae*, which is composed of polysaccharide chains with different molecular weights and contains about 73% polysaccharides, 15% protein, 7% lipids, and inorganic components [4]. Zymosan is not easily degraded; therefore, it can cause oxidative stress after intraperitoneal injection. The overproduction of reactive oxygen species (ROS) could induce apoptosis by activating inflammatory cytokines; then it can cause sustained inflammatory reactions, overly release inflammatory mediators, and produce acute lung injury [4,7].

Many studies have found that excessive production of ROS and an imbalance of the antioxidant defense systems play an important role in the pathogenesis of ALI [8,9,10]. Previous studies have determined that ZY could induce ALI in mice. A danger signal such as tumor necrosis factor-α (TNF-α) could activate the inflammatory body signal pathway, which may be the key target to result in inflammatory response and organ injury [11]. Under pathological conditions, the overproduction of ROS could induce apoptosis by activating inflammatory cytokines such as TNF-α and interleukins (IL-1β and IL-6) [12]. These cytokines are involved in the innate immune response and further cause severe damage to the lung tissues, which ultimately leads to ALI [13]. Furthermore, during ALI, too high inflammatory mediator levels could result in excessive production of free radicals. However, the excessive ROS can be scavenged by antioxidant agents of vitamin C and E, as well as the antioxidant enzymes such as superoxide dismutase (SOD) and catalase (CAT) in vivo [14,15]. Thus, inhibiting inflammation is an important link for the prevention of ALI. Furthermore, previous reports have shown that ROS can produce lipid peroxidation by binding to polyunsaturated fatty acids in the cell membrane, resulting in producing highly cytotoxic malondialdehyde (MDA), which subsequently destroys the stability of the biofilm and increases its permeability, leading to cell damage or necrosis [16,17,18]. Both lipid peroxidation and excessive inflammation response caused by oxidative stress are important reasons of ALI, and the pathological changes of ALI are characterized by oxidative damage, excessive inflammation, and lipid peroxidation [19].

The pathogenesis of and therapeutic drugs for ALI are have been the focus of current research for 30 years [20,21,22,23,24]. In recent years, mushrooms have attracted more and more attention for the dietary nutrition of the bioactive components, and the ancillary products including polysaccharides which have been used as functional foods and represented the basis for the development of new biopharmaceuticals [14,25]. Polysaccharides have gained much attention because of the nutritive and pharmaceutical benefits including antioxidation, anti-inflammatory and anti-tumor effects, immunoregulation, and anticoagulant and organ protection effects [14,26,27], which can be isolated from not only the fruiting body but also mycelium, fermentation broth, and residues [28]. *Pleurotus geesteranus* has recently been gaining popularity, and it is one of the most widely cultivated and rich in biologically active components [28,29]. Based on previous studies by academic researchers, the polysaccharides from the fruiting body of *P. geesteranus* have strong antioxidant and anti-tumor activities [28]. Our previous data have shown the anti-inflammatory, anti-oxidant, and hepatoprotective effects of mycelium polysaccharides from *P. geesteranus* [30]. Moreover, previous studies found that acetylated modification of *Grifola frondosa* polysaccharides could markedly enhance both adjuvant and growth inhibitory effects [31]. However, the effects of acetylated polysaccharides of *P. geesteranus* on ALI have not been reported.

This work was designed to investigate anti-inflammatory and lung protection effects of acetylated *P. geesteranus* polysaccharides (AcPPS) on ALI, and explore the NF-κB signal pathway preliminarily, to identify the underlying mechanisms for the clinical application. Furthermore, the physicochemical properties of AcPPS, such as monosaccharide compositions, molecular weight, and chemical bonds, were also investigated by high-performance gel permeation chromatography (HPGPC), gel permeation chromatography (GPC), Fourier transform infrared spectroscopy (FT-IR), and scanning electron microscopy (SEM).

## 2. Results

### 2.1. Preparation and Yield of AcPPS

**Figure 1:**
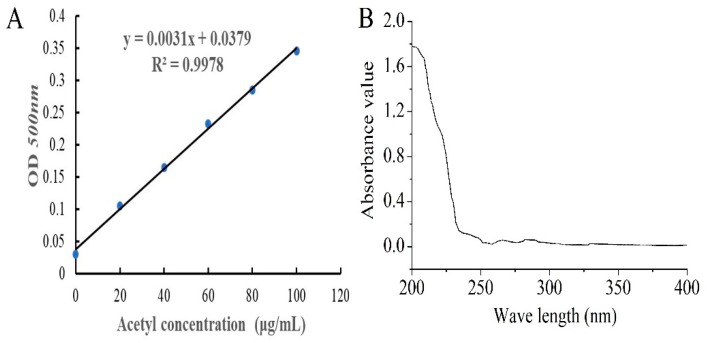
Preparation of acetylated *Pleurotus geesteranus* polysaccharides (AcPPS). (**A**) The standard curve of acetyl content. (**B**) Ultra-violet (UV) analysis.

The standard curve of acetyl content is shown in Figure 1A, and the degree of substitution (DS) of AcPPS was 0.32 according to the equation (y = 0.0031x + 0.0379, R^2^ = 0.9978). The purity of AcPPS was analyzed by ultraviolet (UV) spectrophotometer, and the results are shown in Figure 1B. The lack of obvious absorption at 260 and 280 nm indicated that there were little proteins or nucleic acids in the polysaccharides, and the weak absorption peak of 252–272 nm was caused by a carbonyl group. 

The yield of polysaccharides from *P. geesteranus* (PPS) was 2.54% (calculated by weight of PPS/weight of dry power), and the yield of AcPPS was 39.37% (calculated by weight of AcPPS/weight of PPS). 

### 2.2. Structural Characterization

**Figure 2:**
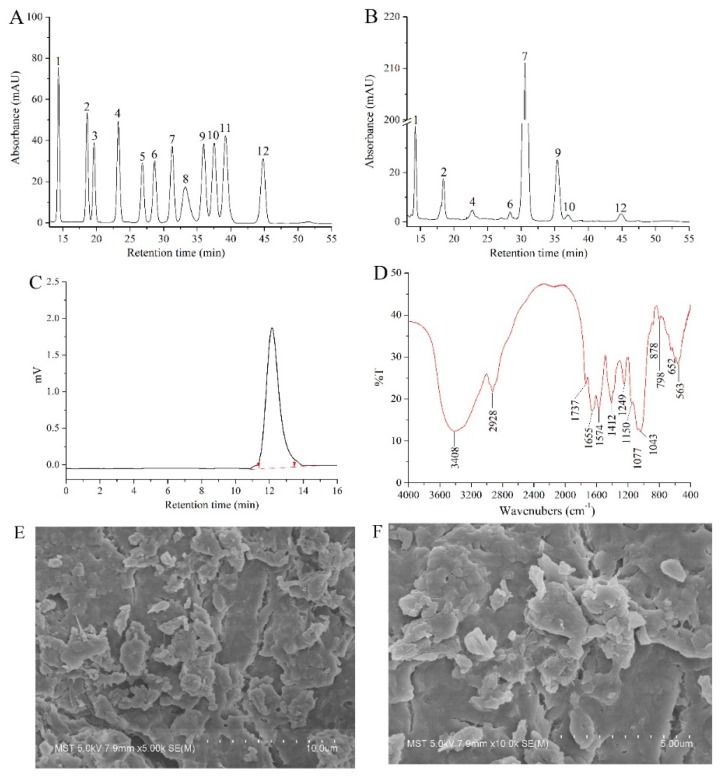
Physicochemical analysis of AcPPS. High-performance gel permeation chromatography (HPGPC) of (**A**) standard sugars and (**B**) AcPPS; (**C**) molecular weights by gel permeation chromatography (GPC); (**D**) Fourier transform infrared spectroscopy (FT-IR) spectra analysis; (**E**) scanning electron microscopy (SEM) images of AcPPS (5000×); (**F**) SEM images of AcPPS (10,000×). Peaks: (1) mannose (Man), (2) ribose (Rib), (3) rhamnose (Rha), (4) glucuronic acid (GlcA), (5) galacturonic acid (GalA), (6) glucosamine (GlcN), (7) glucose (Glc), (8) aminogalactose (GalN), (9) galactose (Gal), (10) xylose (Xyl), (11) arabinose (Ara), and (12) fucose (Fuc).

In this study, the monosaccharide composition of AcPPS was identified by comparing the retention time with that of standard sugars (Figure 2A,B). The AcPPS contained mannose (Man), ribose (Rib), glucuronic acid (GlcA), glucosamine (GlcN), glucose (Glc), galactose (Gal), xylose (Xyl), and fucose (Fuc) with mass percentages of 6.69, 3.76, 1.06, 0.32, 76.96, 9.91, 0.76, and 0.54%.

The AcPPS was analyzed by GPC and monitored with a differential refractive index detector (RID) as described. As shown in Figure 2C, AcPPS has homogeneous polysaccharides with a number-average molar mass (Mn), weight-average molecular weight (Mw), and z-average molecular weight (Mz) of 6279, 9185, and 12,023 Da, respectively.

The FT-IR spectra of AcPPS ranging from 4000 to 400 cm^−1^ are presented in different IR spectra (Figure 2D). AcPPS showed the characteristic bands around 3400 cm^−1^ because of the O-H stretching vibration [27]. The weak band at the peak of 2928 cm^−1^ was C-H stretching vibration for characteristic absorption peaks of sugars, and absorptions at 1574 and 1412 cm^−1^ were the characteristics of C-H vibration, respectively [32]. Additionally, the absorbances at 1737 cm^−1^ and the peak of 1249 cm^−1^ were C=O of esters and C-O stretching vibrations of carbonyl groups, indicating that the AcPPS was successfully acetylated with minor changes of the molecular structure [14,15,33]. Moreover, the band at 1640 cm^−1^ was due to C=O (-COOH) stretching vibration, which was the proof of the presence of uronic acids [34]. Furthermore, there were three absorption peaks of AcPPS within the range of 1000–1200 cm^−1^ indicating the presence of a pyranose ring in polysaccharides [27,32,33,34]. Distinctively, the weak absorption peak at 890 cm^−1^ indicated the existing of β-type glycosidic bond in AcPPS [35].

SEM is a powerful tool to analyze the surface morphology of biomacromolecules and understand their common physical properties. As can be seen in Figure 2E,F, under 5000× and 10,000× magnification, the micro-structure of AcPPS presented an irregular stratified structure with small pores, and the particles were gathered into a block-shaped or granuliform mass with a non-uniform size.

### 2.3. Acute Toxicity Analysis

In the present study, the mice did not exhibit any clinical toxicity symbols even at the highest dose of AcPPS (1500 mg/kg bw). There were no deaths during the post-treatment period (28 days), indicating that AcPPS was practically non-toxic.

### 2.4. Effects of AcPPS on Body Weight and Lung Index

The mice were given an intraperitoneal injection of zymosan paraffin suspension for the first time, and the mice in the early stage of the disease showed dyspnea, lethargy, etc. The body weight changes of all mice before and after intraperitoneal injection with zymosan suspension are shown in Table 1. Before zymosan injection, the difference in body weight in all mice was not significant. However, the body weight in the model control (MC) group declined after ZY-injection, while the body weight in the other mice (AcPPS-intervention) decreased and then increased at different rates after the zymosan injection. On the 25th day, the body weight was decreased by 7.02% in the MC group, while it was decreased by 1.63%, 2.59%, and 3.37% in the H-AcPPS, M-AcPPS, and L-AcPPS groups (high dosage group of AcPPS: H-AcPPS, 800 mg/kg bw; middle dosage group of AcPPS: M-AcPPS, 400 mg/kg bw; low dosage group of AcPPS: L-AcPPS, 200 mg/kg bw) when compared with the body weight on the 22nd day, respectively. The body weight was increased by 3.60%, 3.07%, and 1.99% with the administration of H-AcPPS, M-AcPPS, and L-AcPPS when compared with the body weight on the 25th day, respectively, while the body weight was decreased in the MC group. The results indicate that the slow weight gain and even weight loss in mice was mainly due to excessive inflammation, and AcPPS could alleviate weight reduction in mice induced by zymosan effectively. Furthermore, AcPPS had great potential effects on alleviating organ damage. Briefly, the lung index was decreased by 41.91%, 38.97%, and 30.15% in the H-AcPPS, M-AcPPS, and L-AcPPS groups in comparison with that in the MC group, respectively.

### 2.5. Effects of AcPPS on Oxidative Stress

**Figure 3:**
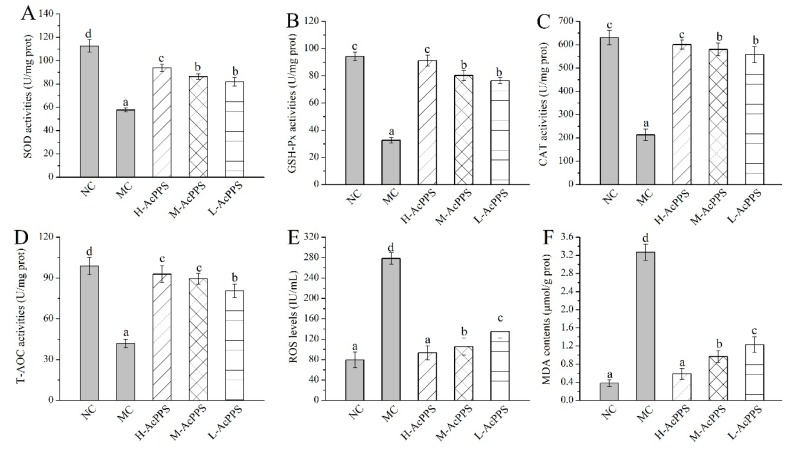
Effects of AcPPS on oxidative stress of lung in acute lung injury (ALI) mice. (**A**) SOD, (**B**) GSH-Px, (**C**) CAT, (**D**) T-AOC, (**E**) ROS, and (**F**) MDA. The values are reported as the Mean ± S.D. (*n* = 10 for each group). Means with the same letter are not significantly different (*p* < 0.05).

The effects of AcPPS on oxidative stress of the lung in ALI mice are demonstrated in Figure 3. Decreases of antioxidant enzyme activities (SOD, glutathione peroxidase (GSH-Px), CAT, and total antioxidant capacity (T-AOC)), as well as increases of the ROS levels and the lipid product contents (MDA), were observed in ALI mice in comparison with the normal control (NC) group to a certain extent, indicating that serious oxidative stress of the lung could be induced by the zymosan injection. Briefly, the SOD, GSH-Px, CAT, and T-AOC activities of lung were increased by 62.66%, 178.62%, 181.33%, and 122.43% in ALI mice treated with H-AcPPS, compared with the MC group (57.76 ± 1.62, 32.69 ± 1.96, 213.39 ± 14.93 and 41.77 ± 3.24 U/mg prot), respectively. Moreover, after the pre-treatment with H-AcPPS, the ROS levels of the lung reached 93.58 ± 13.78 IU/mL with a decrease rate of 66.39% compared with the MC group (278.45 ± 11.32 IU/mL). Additionally, the contents of MDA of the lung reached 0.59 ± 0.12 μmol/g prot with a decrease rates of 81.96% compared with the MC group after the administration of H-AcPPS. The results analysis demonstrated that the acetylated polysaccharide had potential protective effects on protecting the oxidative stress and suppressing lipid peroxidation.

### 2.6. Effects of AcPPS on Lipid Peroxidation

The serum lipid parameters appeared to show apparent abnormality in model control (MC) mice when compared with those in the NC group, showing that lipid peroxidation occurred. As shown in Table 2, the lipid metabolism levels (total cholesterol (TC), triglyceride (TG), low density lipoprotein cholesterol (LDL-C), and very low-density lipoprotein cholesterol (VLDL-C)) were increased, while the high-density lipoprotein cholesterol (HDL-C) level was decreased in ALI mice when compared with the NC group, which is evidence for a disorder of the lipid metabolism. Decreases of TC and TG levels could be observed in the mice treated with AcPPS when compared with the MC group, indicating that supplementation with AcPPS had potential effects on depressing the lipid levels in ALI mice. Additionally, after the gavage administration with H-AcPPS, the HDL-C level was 100.99% higher than that in the MC group (1.01 ± 0.08 mmol/L), while the LDL-C and the VLDL-C levels reached 0.67 ± 0.07 and 0.45 ± 0.02 mmol/L, all being lower than those in the MC group, respectively.

### 2.7. Histopathological Observations

**Figure 4:**
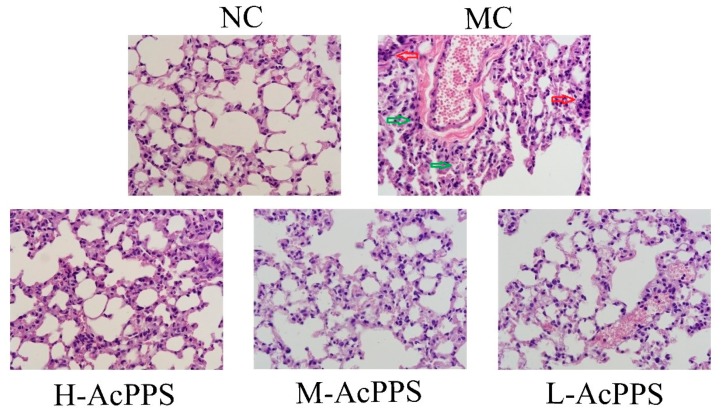
Representative photomicrographs of lung histopathology by H&E staining (600×). Red arrows represent inflammatory cells infiltration. Green arrows represent alveolar collapse.

In the present work, the histopathological examinations of the lung were observed by optical microscope (600×), and the results are shown in Figure 4. Obviously, the normal mice expressed the normal cell morphology and cellular configurations with a normal size of lung, and the alveoli was grid shaped. However, the alveolar septum in the MC group was significantly thickened and fused, and alveolar collapse, with the interstitial neutrophils infiltrating, was identified; the capillary bed was highly dilated and congested, erythrocyte stasis, alveolar hemorrhages, and interstitial expansion occurred, and the alveolar size was different, which was the evidence of the diagnosis of ALI. Fortunately, changes of the structures of the pulmonary lesions caused by zymosan were markedly ameliorated, and the neutrophil accumulation was attenuated when pre-treated with AcPPS at the tested dosages, especially in the H-AcPPS group. The pretreatment with AcPPS, especially the high dosage, showed potential improvement on histopathological observations evidenced by diminutions of necrotic zones against zymosan-induced organic damages.

### 2.8. Effects of AcPPS on Inflammatory Index of BALF

**Figure 5:**
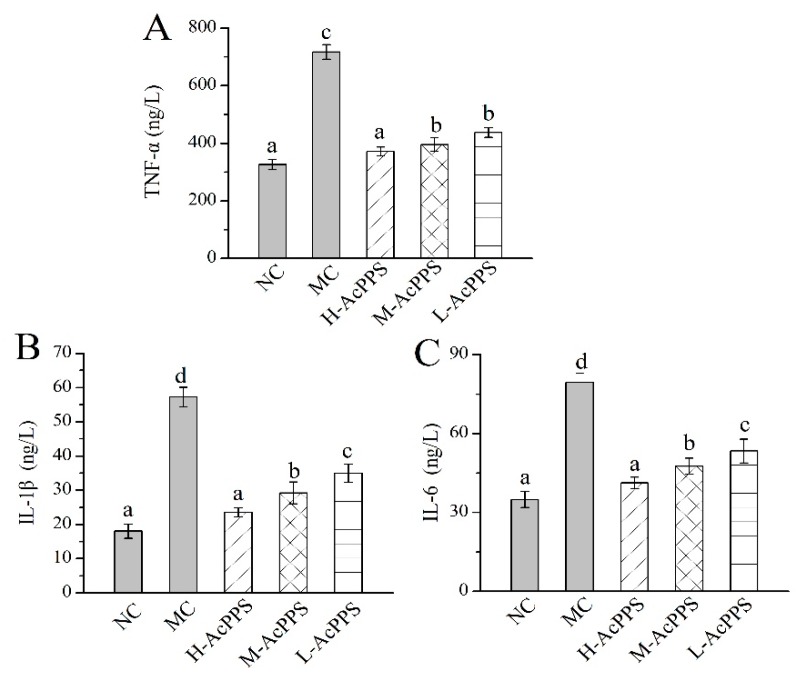
Effects of AcPPS on inflammatory cytokines of bronchoalveolar lavage fluid (BALF) in ALI mice. (**A**) TNF-α, (**B**) IL-1β, and (**C**) IL-6. The values were reported as the Mean ± S.D. (*n* = 10 for each group). Means with the same letter are not significantly different (*p* < 0.05).

The levels of important cytokines in the bronchoalveolar lavage fluid (BALF) were used as biochemical markers for early lung damage. As displayed in Figure 5, the levels of TNF-α, IL-1β, and IL-6 in the MC group were increased 2- to 5-fold compared with those in the NC group, showing that the ALI model was successfully established. Briefly, the TNF-α, IL-1β, and IL-6 levels reached 371.18 ± 16.15, 23.56 ± 1.28, and 41.21 ± 2.19 ng/L with decrease rates of 55.80%, 58.83%, and 48.06% compared with the MC group after the pre-treatment with H-AcPPS, respectively. Apparently, the inflammatory reaction was significantly remitted with the increasing dosage of AcPPS, testifying that AcPPS had potential anti-inflammatory effects on ALI mice.

### 2.9. NF-κB Pathway Detection

**Figure 6:**
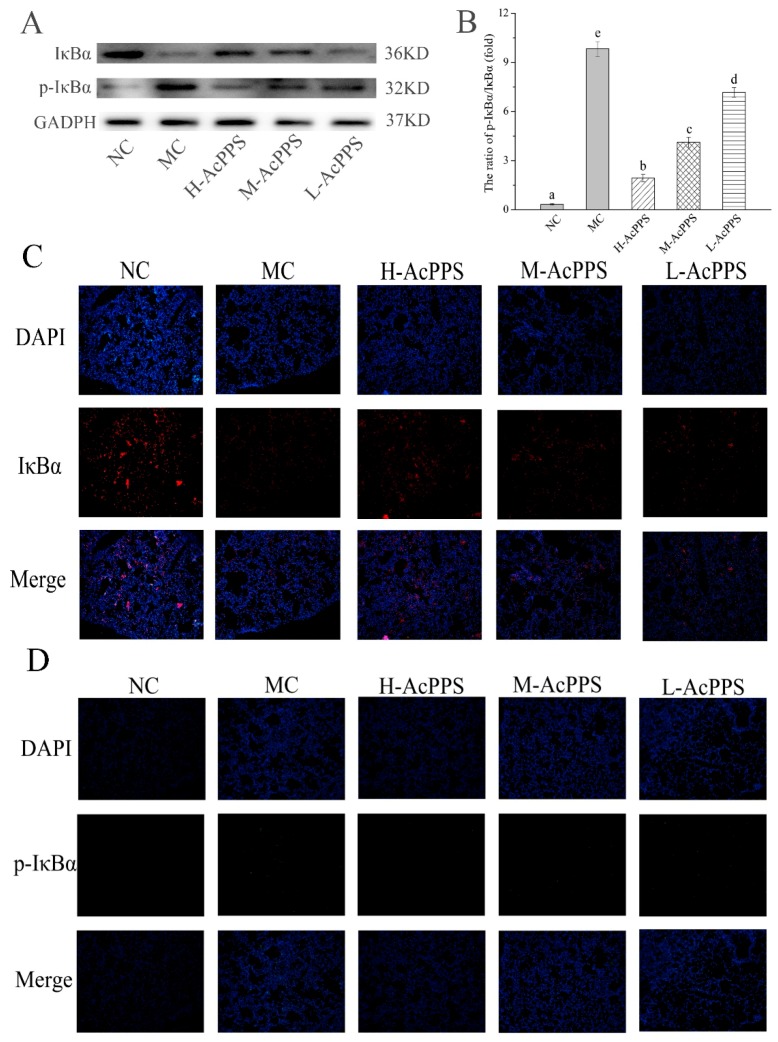
AcPPS adjusted NF-κB signal pathway in mice. (**A**) Effects of AcPPS on the expression levels of p-IκBα and IκBα based on Western blotting assay. (**B**) p-IκBα/IκBα ratios. (**C**) Effects of AcPPS on IκBα level based on immunofluorescence (IF) assay in lung (200×). (**D**) Effects of AcPPS on p-IκBα level based on IF assay in lung (200×).

To examine the role of AcPPS on signal path in the lung of ALI mice, the expression of IκBα and p-IκBα were denoted by Western blot and immunofluorescence (IF) assay. As shown in Figure 6A, the expression of pivotal protein of IκBα was decreased, while p-IκBα was increased in the MC group compared with the NC group. The results demonstrated that the NF-κB pathway was activated. However, the ratios of p-IκBα/IκBα decreased with the intervention of AcPPS, while it increased in the MC group, suggesting that the inhibition of inflammation by polysaccharides might be associated with the NF-κB signal pathway. As shown in Figure 6C,D, the expression level of IκBα was down-regulated, and the p-IκBα level was markedly up-regulated in the MC group based on the IF assay, while the expression level of the pivotal protein returned to the normal level by the regulation of AcPPS, which was consistent with the data based on the Western blotting assay (Figure 6A).

## 3. Discussion

In recent years, acetylated polysaccharides were confirmed to possess significant biological activities, such as antioxidant, anti-inflammatory, cytoprotective, and immune activities and so on [14,36], which has received more and more attention. However, little was known about the acetylated polysaccharides from *P. geesteranus*. Herein, we prepared purified acetylated PPS, and the AcPPS was characterized in our previous work. The data showed that AcPPS contained Man, Rib, GlcA, GlcN, Glc, Gal, Xyl, and Fuc, and it had a weak absorption peak at 252–272 nm, indicating that the polysaccharides were replaced by a carbonyl group. Additionally, the broad peak of AcPPS at around 3400 cm^−1^ was assigned to O-H stretching vibration stretching. A new absorption at around 1737 cm^−1^ corresponded to C=O stretching vibrations of esters, and the absorption at 1249 cm^−1^ was assigned to C-O stretching vibration in carbonyl groups. This is consistent with the results of some other related studies [15,33]. Furthermore, the molecular weight of acetylated polysaccharide was relatively small, and the surface morphology of AcPPS was analyzed. The results showed the particles were gathered in a block-shaped or granuliform mass with small pores. The data indicated that the acetylation modification reduced the molecular weight and changed the structure of polysaccharides, in accordance with the results of acetylated polysaccharides from *Ganoderma atrum* [14]. Therefore, the acetylation of polysaccharides was successful. Moreover, the addition of acetyl groups to polysaccharides increases the water solubility and thus contributes to the development of biological activity.

Under normal physiological conditions, inflammation is beneficial, and it can provide methods for the organism to deal with various injuries. However, poorly regulated and excessive inflammatory response may cause extensive tissue damage and even systemic dysfunction [19,23]. ALI is difficult to treat and has a poor prognosis, which is one of various severe inflammatory diseases [37]. Therefore, it is necessary to elucidate the mechanisms in the development of ALI. Moreover, ZY is a substance derived from the cell wall of the yeast *S. cerevisiae*. It does not introduce exogenous bacteria and endotoxin, which can lead to systemic inflammation or ALI by inducing a wide range of inflammatory mediators [4,38]. The mechanism is that zymosan can cause the inflammatory response to be out of control, over-activate the complement system, and mobilize inflammatory cytokines to participate in the immune response [39]. As a consequence, the mice model of zymosan-induced inflammation has been widely used to study ALI and also for its uniform reagent dosage both at home and abroad [39]. Thus, the ALI model was established in our study.

**Figure 7:**
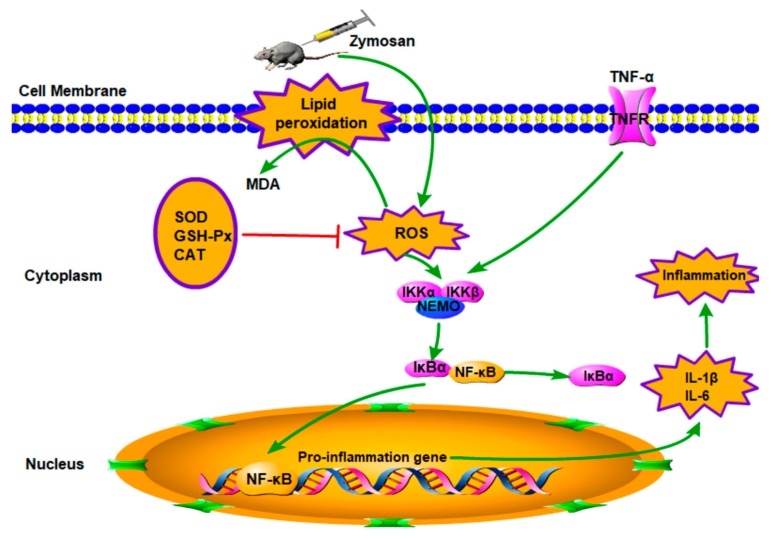
Schematic diagram of signal pathway related to anti-inflammatory effects of AcPPS on ALI.

The mechanisms of ALI have not yet been fully elucidated; however, ALI is generally believed to be caused by cascade uncontrolled systemic inflammatory response, and the role of inflammatory mediators in pathophysiology has attracted more and more attention. Excessive inflammatory response and oxidative–antioxidant balance disorders are the pathological basis of organ damage [21,22,23]. As shown in Figure 7, on the one hand, oxygen free radicals are considered to be an important factor leading to the pathogenesis [39]. The ROS produced by normal cells does not cause cellular damage under normal physiological conditions [40]. The formation of ROS cannot be inhibited if the function of scavenging free radical system is reduced, including antioxidant enzymes such as SOD and CAT, and the amounts of free radicals far exceeds the scavenging system; thus, the balance of oxidation–antioxidation is destroyed [10,18,41]. The excess of ROS can lead to oxidant stress injury. SOD is one of the important antioxidant enzymes which can eliminate endogenous ROS and prevent its induced oxidative stress, and T-AOC is one of the significant indicators of the body’s antioxidant effect [12]. Moreover, the ROS-induced oxidant stress, which can lead to free radical chain reactions with unsaturated fatty acids, may generate toxic lipid intermediates and modify the integrity of cellular membranes and damage cells, inducing apoptosis and necrosis. MDA is a degradation product of lipid peroxidation, which is a well-known indicator for measuring oxidative damage and its content can reflect the degree of lipid peroxidation in the body [42]. Therefore, oxidative stress plays an important role in ALI. In the present study, an ALI model was established, and ZY significantly increased levels of MDA and ROS in the MC group compared to the normal group in lung. However, AcPPS reduced the level of MDA and ROS, especially H-AcPPS. Concomitantly, we also found that the activities of SOD, T-AOC, and MDA were decreased in the MC group, but increased by the treatment with AcPPS. The results were in accordance with the reported by Chen et al. [14]. The results indicated that the oxidative stress was inhibited by AcPPS *via* increasing SOD, T-AOC, and MDA activities and decreasing ROS and MDA levels, suggesting that the AcPPS were novel bioactive compounds responsible for the antioxidant effects. Furthermore, the free radical can attack the lipids of biofilms and tissues because of a strong reaction ability, causing lipid peroxidation and disorder of the lipid metabolism [40]. The increased blood lipid levels are due to lipid peroxidation altering the permeability of cell membrane and increasing the levels of TC and TG levels in serum. Briefly, TC can be transferred from the liver to peripheral tissues by HDL-C *via* the “cholesterol transport” pathway, participating in blood circulation, while the abnormal changes of LDL-C and VLDL-C can result in oxidative stress [43]. In our study, AcPPS administration showed effects on lowering TC, TG, LDL-C, and VLDL-C levels and elevating HDL-C levels in ALI mice, suggesting that AcPPS had potential effects on improving the dyslipidemia associated with zymosan toxicity. All of the dates were in keeping with the conclusion of Xie et al. and Zhao et al. [4,39].

On the other hand, ALI is mainly caused by the uncontrolled inflammatory response of the body. The participation of various inflammatory mediators is the key to the pathogenesis [37,39]. In the process of acute lung injury caused by zymosan, immune cells can be activated and release a lot of pro-inflammatory cytokines such as TNF-α, IL-1β, and IL-6, which can lead to an inflammatory cascade, resulting in lung damage. The important inhibitor IκBα was phosphorylated and then degraded when the NF-κB signal pathway was activated, and the remaining inflammatory transcription factor NF-κB enters the nucleus and binds to promoters or enhancers of various inflammatory factors. TNF-α is the first inflammatory mediator that induces ALI and is the initiating factor leading to the cascade of inflammatory mediators, and it can directly lead to apoptosis or even death [44,45]. Moreover, a large amount of TNF-α is secreted after ZY-injection, which can stimulate the production of IL-1β and IL-6, while IL-6 is considered to be an important indicator of the persistence of inflammation [4]. In addition, TNF-α can also release a large amount of oxygen free radicals to further aggravate organ damage. Ultimately, these inflammatory mediators initiated transcription, causing a variety of pro-inflammatory cytokines to be produced in large quantities and in turn continuing to activate the NF-κB signal pathway, forming a positive feedback adjustment [18,43]. In our study, high levels of inflammatory cytokines such as TNF-α, IL-1β, and IL-6 have been detected inflammatory BALF after ZY-injection. The levels of TNF-α, IL-1β, and IL-6 of BALF were increased significantly in the MC group, compared with the NC group; meanwhile, the levels of the inflammatory cytokines were reduced in the AcPPS treatment group relative to the MC group. Moreover, the expression level of IκBα was markedly down-regulated, and the up-regulated level of p-IκBα in the MC group based on Western blotting assay and immunofluorescence assay, while the expression level of the pivotal protein returned to the normal level by the regulation of AcPPS. The application of AcPPS succeeded to inhibit inflammatory cytokines release, suggesting a relief of inflammatory symptoms. Our data were in accordance with those reported by Yang et al. [15]; thus, we could speculate that AcPPS might protect the lung against senescence by inhibiting the NF-κB signal pathway based on the changes in protein levels (IκBα and p-IκBα).

## 4. Materials and Methods

### 4.1. Chemicals and Reagents

Diagnostic kits for investigating the activities of MDA, SOD, CAT, and total T-AOC were from Nanjing Jiancheng Bioengineering Institute (Nanjing, China). The enzyme-linked immunosorbent assay (ELISA) diagnostic kits for ROS, TNF-α, IL-1β, and IL-6 investigations were supplied by Jiangsu Meibiao Biological Technology Company, Limited (Jiangsu, China). The primary antibodies rabbit polyclonal anti-IκBα, anti-p-IκBα, rabbit anti-GAPDH, and the secondary antibody goat anti-rabbit IgG-HRP used in the signal pathway were purchased from Absin Bioscience Inc. (Shanghai, China). Cyanine3 (CY3) conjugated Goat Anti-rabbit IgG and Fluorescein isothiocyanate (FITC) conjugated Goat Anti-rabbit IgG used in the IF were supplied by Servicebio Bioscience Inc. (Wuhan, China). The standard monosaccharaides, including Man, Rib, rhamnose (Rha), GlcA, galacturonic acid (GalA), GlcN, Glc, aminogalactose (GalN), Gal, Xyl, arabinose (Ara), Fuc, and a series of standard dextran were purchased from Sigma Chemicals Company (St. Louis, MO, USA). All other reagents and chemicals used in the present work were analytical grade and provided by local chemical suppliers in China.

### 4.2. Materials and Methods

The fruiting body of *P. geesteranus* was provided by Quanrun Edible Fungus Production Base (Linyi, China).

### 4.3. Preparation and Purification of AcPPS

#### 4.3.1. Preparation of AcPPS

The dried powder of *P. geesteranus* (5000 g) was extracted with boiling water for 3 h with a solid–liquid ratio of 1:3. After centrifugation at 3000× *g* for 15 min, the supernatant was precipitated with ethanol (1:3, 95%, *v/v*) at 4 °C overnight. The resulting precipitate was collected by centrifugation (3600× *g*, 10 min) and deproteinated with the method of Sevag (chloroform-butanol, 5:1 *v/v*) [46,47]. After dialysis with distilled water for 48 h and being freeze-dried (Labconco, Kansas City, MO, USA), the precipitate was considered as PPS.

Acetylation of PPS was performed by the reported method with modifications [14]. Briefly, PPS (0.5 g) was dissolved in distilled water (20 mL), and the pH was adjusted to 9.0 with 1 mol/L NaOH under stirring. Then the homogeneous solution was kept at room temperature for 20 min, and 2 mL of acetic anhydride was added to obtain acetylated derivatives. NaOH (1 mol/L) was subsequently added to each mixture to maintain the pH at 8.0-11.0 (30°C). After 3 h, the reaction was stopped with 1 mol/L HCl, and the pH was adjusted to 7.0. The resultant solution was dialyzed with distilled water for 48 h and then for another 48 h in a dialysis tube (Solarbio, Beijing Solarbio Science & Technology Co., Ltd.) with a cut-off molecular weight (3500 Da), concentrated, and freeze-dried (Beili, Beijing, China and SCIENTZ, Ningbo, China); then AcPPS was obtained. After dialysis with distilled water, the sample was concentrated and lyophilized for further experiments.

#### 4.3.2. Standard Curve Plotting of Acetyl Content

The DS was measured using the method reported by Chen et al. [14] with a slight modification. β-D-glucose pentaacetate (348.9 mg) was dissolved into 10 mL ethyl alcohol adequately, and then the solution was kept at a constant volume and shook up in 50 mL volumetric flask. The aforementioned β-D-glucose pentaacetate stock solution (1, 2, 3, 4, 5, 6 mL) was removed from the volumetric flask (25 mL) and used at constant volume, respectively. The above solution (5 mL), 5 mL hydroxylamine hydrochloride (0.1 mol/L), 5 mL sodium hydroxide solution (1.5 mol/L), 5 mL hydrochloric acid solution (2 mol/L), and 10 mL ferric chloride (0.37 mol/L) were added successively at constant volume (50 mL) with deionized water. Finally, the absorbance at 500 nm was measured based on the series of concentrations of acetyl.

#### 4.3.3. The DS Assay of AcPPS

A brown volumetric flask containing 1 mL sample solution (1 mg/mL), 5 mL hydroxylamine hydrochloride (0.1 mol/L), and 5 mL sodium hydroxide solution (0.15 mol/L) was kept for 20 min. Above solution was neutralized with 5 mL hydrochloric acid solution (2 mol/L), and ferric chloride (0.37 mol/L, 10 mL) was added after 20 min. The absorbance at 500 nm was measured after adjusting to constant volume (50 mL) with deionized water. The DS was expressed as:

W (%) = W1/W2 × 100(1)

DS = 162 × W/(4300 − (43-1) × W)(2)

where W is the mass fraction of the acetyl (%), W2 is the quality of AcPPS (mg), W1 is the acetyl quality of AcPPS (mg), 162 is the relative molecular mass of each polysaccharide residues, 43 is the relative molecular mass of acetyl, 1 is the relative atomic mass of hydrogen.

#### 4.3.4. UV Analysis

The ultraviolet spectrum of the polysaccharide samples with a final concentration of 0.1% in 1 cm quartz sanding trail pool was recorded with an UV-visible spectrophotometer (UV-1800, SHIMADZU, Kyoto, Japan) in the range of 200–400 nm.

### 4.4. HPGPC, GPC, FT-IR Spectroscopy, and SEM Analysis

The monosaccharide compositions were determined with pre-column derivatization by HPLC (Agilent Technologies, Alpharetta, GA, USA) equipped with a chromatographic column of Xtimate C18 (4.6 m × 200 mm × 5 μm). The AcPPS (10 mg) was hydrolyzed with trifluoroacetic acid (2 mol/L, 2 mL) in an ampere bottle at 110 °C for 8 h and re-dissolved with distilled water (2 mL). The hydrolyzed samples and the monosaccharide standards were labeled with polymethylpentene (PMP) by adding 250 μL of 0.6 mol/L NaOH and 500 μL of 0.4 mol/L PMP-methanol solution. The mixture was reacted at 70 °C for 1 h and then neutralized with 500 μL of 0.3 mol/L HCl and extracted with 1 mL chloroform three times. The liquid supernatant was analyzed using a HPLC system comparison with the standard sugars of Man, Rib, Rha, GlcA, GalA, GlcN, Glc, GalN, Gal, Xyl, Ara, and Fuc. The chromatographic temperature was 30 °C, and the mobile phase was a mixture of phosphate buffer solution (0.05 mol/L, pH 6.7) with a ratio of 87:13 (*v/v*). The sample volume was 20 μL with a rate of 1.0 mL/min, and the detector wavelength was 250 nm.

The molecular weights of AcPPS were estimated by GPC and a refractive index detector (LC20+RID20A, Agilent Technologies, USA). The sample (100 µL) was injected into the column using deionized water as the mobile phase with a flow rate of 1.0 mL/min, and the column temperature was maintained at 30 °C. A series of standard dextran (T-200, T-100, T-70, T-50, T-40, T-20, T-10, and T-5, Sigma) were used to make the calibration curve, and the molecular weight was analyzed by Agilent GPC software.

The AcPPS samples (1 mg) were mixed with KBr powder (100–200 mg) and then pressed into pellets for infrared spectral analysis within a range of 4000–400 cm^−1^. The FT-IR spectrum of the polysaccharide was measured by a spectrophotometer (Nicolet 8700, Thermo Nicolet Corporation, Wisconsin, USA).

The morphologies of AcPPS were obtained by using cold field emission scanning electron microscopy (S4800, Hitachi Limited, Tokyo, Japan). The powdered sample was directly mounted on a metal stub and then sputtered with gold powder. Finally, the samples were observed with 5000- and 10,000-fold magnification at 7.0 kV under a high-vacuum condition.

### 4.5. Acute Toxicity Experiment

The acute toxicity experiment was processed using the method reported by Zhang et al. [48]. Forty Kunming strain mice (male, 8 weeks old, 20 ± 2 g) were obtained from Taibang Biologic Products Limited Company (Taian, China) and randomly divided into four groups (10 mice in each group). The mice in the control group received free access to food and water, and the mice in the experimental groups orally received AcPPS (dissolved in deionized water) with the dose of 500, 1000 or 1500 mg/kg bw for 28 consecutive days. The mice were observed continuously for normal appearance or behavioral changes during the whole feeding period.

### 4.6. Animal Experiments

The Kunming strain mice (male, 8 weeks old, 20 ± 2 g) were housed in cages under controlled conditions of 12 h light/dark cycles at 22 ± 2 °C and 50%–55% humidity with free access to water and standard food. All experiments were performed in accordance with the Regulations of Experimental Animal Administration issued by the State Committee of Science and Technology of the People’s Republic of China.

After adapting to the environment for one week, all mice were randomly divided into five groups (ten mice per group) including NC group, MC group, and three dosage groups of AcPPS (dissolved in deionized water), including high (H-AcPPS, 800 mg/kg bw), middle (M-AcPPS, 400 mg/kg bw), and low (L-AcPPS, 200 mg/kg bw) dosage. In the NC group, mice were intraperitoneally injected with sterile saline, while the three AcPPS groups’ mice were intragastrical treated respectively for four consecutive weeks. On the 22nd day, all mice except those in the NC group were intraperitoneal injected with zymosan suspension solution (500 mg/kg bw, suspended in saline solution) for one time to induce ALI [37]. Briefly, the zymosan was suspended by high-frequency vibration of saline solution, and the suspension was sterilized at 100 °C for 60 min. During the experiment, all mice were weighed on the 22nd, 25th, and 28th day, respectively. On the 28th day, all mice were fasted overnight and sacrificed via anesthetics.

The collection of BALF was performed with 1 mL sterile PBS (pH 7.2) three times (total volume 3 mL). After centrifugation (3000× *g*, 4 °C, 15 min), the supernatants of BALF were kept at −80 °C for further cytokine analysis. The contents of TNF-α, IL-1β, and IL-6 in BALF were measured using ELISA diagnostic kits.

The serum was separated from blood samples by centrifugation (14,000× *g*, 10 min). The TC, TG, HDL-C, LDL-C, and VLDL-C levels were measured using an automatic biochemical analyzer (ACE, USA).

The lung was rapidly excised, weighed, and homogenized (1:9, w/v) in phosphate buffer solution (0.2 mol/L, pH 7.4). After centrifugation (5000× *g*) at 4 °C for 20 min, the supernatants were stored at 4 °C for further biochemical analysis. The activities of SOD, GSH-Px, CAT, and T-AOC, as well as the contents of MDA in lung were analyzed using commercial kits according to the instructions. In addition, the level of ROS lung was measured using ELISA diagnostic kits. The main steps of ROS are as follows: 1. Standard, sample diluent; 2. Add the standard and sample diluent, and incubate for 30 min at 37 °C; 3. Wash 5 times, add HRP-conjugate reagent, and incubate for 30 min at 37 °C; 4. Wash 5 times, add chromogen solutions A and B, and incubate for 10 min at 37 °C; 5. Add the stop solution; 6. Read the absorbance at 450 nm within 15 min; 7. Calculate: Assay range: 20 IU/mL–500 IU/mL. The other steps of the ELISA kits are similar to those for ROS.

Sample preparation of Western blot was prepared by homogenization in ice-cold lysis buffer, supplemented with protease/phosphatase inhibitor cocktails. The lysates were centrifuged at 10,000× *g* at 4 °C for 10 min. The protein was mixed with loading buffer, denatured in boiling water for 10 min, and the supernatant was collected. The protein expression levels of IκBα, p-IκBα, and GAPDH in the inflammatory body signal pathway were detected by Western blotting to detect the degree of inflammatory response.

The fresh lung was immediately prepared for hematoxylin-eosin (H&E) staining histological and morphological analysis. All of the sections were photographed under a light microscope to assess the degree of the lung injury (600× magnification). Immunofluorescence staining was analyzed by CY3 (IκBα) and FITC (p-IκBα) as the secondary antibodies under a fluorescence microscope (200× magnification).

### 4.7. Statistical Analysis

All the data were shown as the Mean ± S.D. (Standard Deviation). Significant differences between the experimental groups were determined using one-way ANOVA followed by Tukey’s tests (SPSS version 19.0, International Business Machines Corporation, New York, NY, USA), and *p* < 0.05 was considered a statistically significant difference.

## 5. Conclusions

The findings successfully presented the acetylation of polysaccharides which had small molecular weight. The AcPPS from *P. geesteranus* possessed potential antioxidation and lung-protective effects against ALI *via* inhibiting the NF-κB signal pathway and might be suitable as natural food for the prevention or remission of ALI.

## Figures and Tables

**Table 1:** Body weight, lung weight, and lung index of all mice after zymosan-infection.

Groups	Body Weight (g)			Lung Weight (g)	Lung Index (%)
	Treatment Time by Zymosan 1 d	Treatment Time by Zymosan 4 d	Treatment Time by Zymosan 7 d		
NC	42.78 ± 0.24 ^a^	43.40 ± 0.19 ^d^	44.50 ± 0.14 ^d^	0.33 ± 0.09 ^a^	0.74 ± 0.09 ^a^
MC	42.60 ± 0.26 ^a^	39.61 ± 0.17 ^a^	38.97 ± 0.23 ^a^	0.53 ± 0.07 ^c^	1.36 ± 0.12 ^c^
H-AcPPS	42.37 ± 0.29 ^a^	41.68 ± 0.14 ^c^	43.18 ± 0.16 ^c^	0.34 ± 0.02 ^a^	0.79 ± 0.07 ^a^
M-AcPPS	42.16 ± 0.12 ^a^	41.07 ± 0.18 ^b^	42.33 ± 0.25 ^b^	0.35 ± 0.04 ^a^	0.83 ± 0.14 ^a^
L-AcPPS	42.68 ± 0.15 ^a^	41.24 ± 0.23 ^c^	42.06 ± 0.14 ^b^	0.40 ± 0.03 ^b^	0.95 ± 0.11 ^b^

The values were reported as the Mean ± S.D. (*n* = 10 for each group). Different letters indicate statistically significant differences, while the same letter indicates statistically insignificant differences (*p* < 0.05).

**Table 2:** Effects of AcPPS on lipid peroxidation in ALI mice.

		NC	MC	H-AcPPS	M-AcPPS	L-AcPPS
Serum lipid parameters	TC (mmol/L)	1.36 ± 0.11 ^a^	2.28 ± 0.15 ^c^	1.47 ± 0.08 ^a^	1.53 ± 0.12 ^a^	1.87 ± 0.09 ^b^
	TG (mmol/L)	0.75 ± 0.02 ^a^	1.83 ± 0.09 ^d^	0.80 ± 0.05 ^a^	0.97 ± 0.08 ^b^	1.06 ± 0.07 ^c^
	HDL-C (mmol/L)	2.16 ± 0.15 ^c^	1.01 ± 0.08 ^a^	2.03 ± 0.11 ^c^	1.95 ± 0.09 ^b^	1.78 ± 0.07 ^b^
	LDL-C (mmol/L)	0.55 ± 0.06 ^a^	1.71 ± 0.12 ^d^	0.67 ± 0.07 ^a^	0.91 ± 0.05 ^b^	1.18 ± 0.15 ^c^
	vLDL-C (mmol/L)	0.34 ± 0.05 ^a^	1.26 ± 0.11 ^d^	0.45 ± 0.02 ^a^	0.61 ± 0.09 ^b^	0.78 ± 0.04 ^c^

The values were reported as the Mean ± S.D. (*n* = 10 for each group). Different letters indicate statistically significant differences, while the same letter indicates statistically insignificant differences (*p* < 0.05).

## Data Availability

The data used to support the findings of this study are available from the corresponding author upon request.

## Abbreviations

AcPPS	Acetylated *Pleurotus geesteranus* polysaccharides
ALI	Acute lung injury
BALF	Bronchoalveolar lavage fluid
GalN	Aminogalactose
Ara	Arabinose
CAT	Catalase
DS	Degree of substitution
ELISA	Enzyme-linked immunosorbent assay
FT-IR	Fourier transform infrared spectroscopy
Fuc	Fucose
Gal	Galactose
GalA	Galacturonic acid
GSH-Px	Glutathione peroxidase
GPC	Gel permeation chromatography
GlcN	Glucosamine
Glc	Glucose
GlcA	Glucuronic acid
HDL-C	High-density lipoprotein cholesterol
H&E	Hematoxylin-eosin
HPGPC	High-performance gel permeation chromatography
IF	Immunofluorescence
IL	Interleukins
LDL-C	Low density lipoprotein cholesterol
MDA	Malondialdehyde
Man	Mannose
MC	Model control
MODS	Multiple organ dysfunction syndrome
NC	Normal control
Mn	Number-average molar mass
PMP	Polymethylpentene
PPS	Polysaccharides from *P. geesteranus*
ROS	Reactive oxygen species
Rha	Rhamnose
Rib	Ribose
SEM	Scanning electron microscope
SOD	Superoxide dismutase
SIRS	Systemic inflammatory response syndrome
T-AOC	Total antioxidant capacity
TC	Total cholesterol
TG	Triglyceride
TNF-α	Tumor necrosis factor-α
UV	Ultraviolet
VLDL-C	Very low-density lipoprotein cholesterol
Mw	Weight-average molecular weight
Xyl	Xylose
Mz	Z-average molecular weight
ZY	Zymosan
ZIGI	Zymosan-induced generalized inflammation
